# Organotin Exposure and Vertebrate Reproduction: A Review

**DOI:** 10.3389/fendo.2018.00064

**Published:** 2018-03-01

**Authors:** Julia Fernandez Puñal de Araújo, Priscila Lang Podratz, Eduardo Merlo, Isabela Valim Sarmento, Charles Santos da Costa, Oscar Mauricio Santamaria Niño, Rodrigo Alves Faria, Leandro Ceotto Freitas Lima, Jones Bernardes Graceli

**Affiliations:** ^1^Department of Morphology, Federal University of Espírito Santo, Vitória, Brazil

**Keywords:** organotin compounds, reproduction, vertebrates, endocrinology, environmental pollutants

## Abstract

Organotin (OTs) compounds are organometallic compounds that are widely used in industry, such as in the manufacture of plastics, pesticides, paints, and others. OTs are released into the environment by anthropogenic actions, leading to contact with aquatic and terrestrial organisms that occur in animal feeding. Although OTs are degraded environmentally, reports have shown the effects of this contamination over the years because it can affect organisms of different trophic levels. OTs act as endocrine-disrupting chemicals (EDCs), which can lead to several abnormalities in organisms. In male animals, OTs decrease the weights of the testis and epididymis and reduce the spermatid count, among other dysfunctions. In female animals, OTs alter the weights of the ovaries and uteri and induce damage to the ovaries. In addition, OTs prevent fetal implantation and reduce mammalian pregnancy rates. OTs cross the placental barrier and accumulate in the placental and fetal tissues. Exposure to OTs *in utero* leads to the accumulation of lipid droplets in the Sertoli cells and gonocytes of male offspring in addition to inducing early puberty in females. In both genders, this damage is associated with the imbalance of sex hormones and the modulation of the hypothalamic–pituitary–gonadal axis. Here, we report that OTs act as reproductive disruptors in vertebrate studies; among the compounds are tetrabutyltin, tributyltin chloride, tributyltin acetate, triphenyltin chloride, triphenyltin hydroxide, dibutyltin chloride, dibutyltin dichloride, diphenyltin dichloride, monobutyltin, and azocyclotin.

## Introduction

Organotins (OTs) are organometallic compounds that are widely used in industry, such as in the manufacture of plastics, pesticides, paints, and others (1, 2). Despite being easily degraded in the environment, several studies have shown the toxicological effects in different trophic levels of the food chain (3, 4). In 2008, the World Health Organization decreed a ban on the use of OTs in paints on vessels. However, many countries did not adopt this ban. OTs are classified as endocrine-disrupting chemicals (EDCs), leading to inappropriate endocrine system functioning in various species (5, 6). Thus, their exposure can cause damage, sometimes irreversibly, such as the process of *imposex* in which female gastropods develop male sex organs (3). For humans and other vertebrates, the major route of OTs exposure is by the intake of contaminated seafood, and studies evaluating their toxicological risks are limited (7–11). OTs impair reproductive functioning, and the damage is associated with the imbalance of sex hormones and with improper modulation of the hypothalamic–pituitary–gonadal axis function of rodents (12–14). Here, we report that OTs act as reproductive disruptors in vertebrate studies (Table 1); among them are tetrabutyltin (TeBT), tributyltin chloride (TBTCl), tributyltin acetate (TBTAc), triphenyltin chloride (TPTCl), triphenyltin hydroxide (TPTOH), dibutyltin chloride (DBTCl), dibutyltin dichloride (DBTCl2), diphenyltin dichloride (DPTCl2), monobutyltin (MBT), and azocyclotin.

**Table 1:** Summary of vertebrate reproductive changes induced by OTs.

	Animal models/dose/OTs						
	Fish		Frog	Rodents		Monkey	Reference
	(0.01–25 µg/kg)		(0.5 µg to 0.5 g/L)	(100 ng to 125 mg/kg)		(2.5–3.8 mg/kg)	
	TriOTs	Azocyclotin	Azocyclotin	TriOTs	DiOTs	DiOTs	
**Adult male**							
Organ weights (g/bw)							
Testes, epididymis	NR	NR	NR	↓	NR	NR	(20, 21)
Prostate, seminal vesicle	NR	NR	NR	↓	NR	NR	(21)
Sn accumulation	NR	NR	NR	↑	NR	NR	(22)
Histopathology							
Spermatocytes/spermatids	NR	↑	NR	↓	NR	NR	(20, 24)
Sperm viability/number	↓	↓	NR	↓	NR	NR	(20, 23, 24)
Seminiferous tubules	NR	NR	NR	↑/↓ Lumen	NR	NR	(21, 22)
Testes	Fibrosis	NR	NR	Edema	NR	NR	(15, 22)
Leidig cells (number)	NR	NR	NR	↓	NR	NR	(22)
Sex hormones	↓ FSH	NR	↑ T	↑/↓ LH, ↓ T	NR	NR	(13, 15, 24, 26, 27)

***In utero*effect**							
Cryptorchidism	NA	NA	NA	↔	NR	NR	(12)
Preputial separation	NA	NA	NA	↔	NR	NR	(54)
Testes, epididymis and prostate weight	NA	NA	NA	↓/↔	NR	NR	(12, 54)
Gonocytes, Sertoli cells (number)	NA	NA	NA	↓	NR	NR	(55)
Spermatid and sperm (number)	NA	NA	NA	↓	NR	NR	(12, 54)
Sperm motility	NA	NA	NA	↓/↔	NR	NR	(12, 54)
Sex hormones	NA	NA	NA	↑T, ↑LH, ↓E2	NR	NR	(12, 53, 54)

**Adult female**							
Estrous cyclicity	NR	NR	NR	Impaired	NR	NR	(14, 31)
Ovary weight (g/bw)	NR	↓	↓	↓/↑	NR	NR	(21, 24, 27, 29, 32)
Sn accumulation	NR	NR	NR	↑	NR	NR	(14)
Histopathology							
Ovarian follicles	NR	NR	NR	↑ Apoptosis, ↑ Atretic	NR	NR	(14, 31, 34)
Folliculogenesis	NR	Impaired	NR	↓ Mature follicles	NR	NR	(14, 24, 31, 37)
Sex hormones	NR	↑T, ↓E2	NR	↑/↓ E2, ↑T	NR	NR	(14, 24, 29, 31)

***In utero*effect**							
Vaginal opening	NA	NA	NA	↑/↓	NR	NR	(26, 56)
Estrous cyclicity	NA	NA	NA	Impaired	NR	NR	(26, 56)
Ovary morphology	NA	NA	NA	Impaired	NR	NR	(55)

**Fertility**							
Loss pre- and postimplantation	NR	NR	NR	↑	↑	↑	(40–43)
Number of live fetuses	↓	NR	NR	↑	NR	NR	(41, 45)
Sex ratio (female/male)	↔/↑/↓	NR	NR	↔	NR	↔	(19, 23, 41, 43–45)
Litter size	NR	NR	NR	↓/↔	NR	NR	(39, 41)
Hatchability/egg viability	↔/↓	NR	NR	NR	NR	NR	(19, 44)

*OTs, organotins; TriOTs, triorganotins (tributyltin chloride, tributyltin acetate, triphenyltin chloride, triphenyltin hydroxide); DiOTs, diorganotins (dibutyltin chloride, dibutyltin dichloride, diphenyltin dichloride); Sn, tin; ↑, increased; ↓, decreased; ↔, unchanged or similar to control; NR, not reported; NA, not applicable; bw, body weight; LH, luteinizing hormone; T, testosterone; FSH, follicle-stimulating hormone; E2, estrogen; Fish, zebrafish (Danio rerio), rockfish (Sebastiscus marmoratus), and Oryzia latipes; frog, Xenopus laevis, Rodents, rats and mice; monkey, Macaca fascicularis*.

## Reproductive Toxicology

### Male Reproductive Function

Several studies have evaluated OT exposure in male vertebrates, highlighting the dose-dependent impairment by OTs in different experimental models (15–19). Male mice at postnatal day (PND) 21 exposed to TBTCl at concentrations of 0.5, 5, and 50 µg/kg for 3 days presented a reduction in testis weight (20). There was also a decrease in testis weight in Swiss Webster mice on PND 15 exposed to a dose of 15 mg/kg/30 days of TPTCl as well as a reduction in epididymis, prostate, and seminal vesicle weight (21). Mitra et al. (22) reported that TBTCl exposure for 3 days at doses of 10–30 mg/kg caused an accumulation of tin in rat testes. Thus, the OTs were able to accumulate in the male reproductive tract, leading to morphofunctional abnormalities.

Studies have reported a consensus that OTs are very harmful to vertebrate reproduction and the quality of spermatozoa (20, 23, 24). A reduction in the numbers of spermatocytes and spermatids as well as sperm viability and an increase in abnormal gametes were observed in male rats after exposure to TBTCl in a dose-dependent manner (20). Similar data were observed in zebra fish (*Danio rerio*); when exposed to different doses of TBTCl from the first day of incubation of the eggs to PND 70, they exhibited effects such as reduced or completely lost sperm motility, absence of flagella, and the presence of only abnormal spermatozoa in semen (23). Similarly, zebra fish exposed to 0.09 and 0.45 µg/L of azocyclotin presented a reduction of 21.4 and 58.1% in the number of spermatozoa (24). However, there was an increase in spermatocytes with exposure to azocyclozine at levels of 0.09 (17.5%) and 0.45 µg/L (63.8%) in these fish at 5–6 months of age (24). Several histological studies have reported that OTs dramatically affect the reproductive apparatus cells in vertebrates (15, 21, 22, 25). Exposure to doses of TBTCl (10, 20, 30 mg/kg) for 1,100–1,300 h affected spermatogenesis, increased the lumen size of the seminiferous tubules, and caused testicular interstitial edema along with evident Leydig cell loss in male rats (22). The seminiferous tubule in male mice that received doses of TPTCl ≥3.75 mg/kg body weight (bw)/day presented a smaller tubule diameter and germinal epithelial reduction, suggesting that TPTCl exposure impaired spermatogenesis (21). Severe interstitial fibrosis was also observed in the interlobular septum of the testis with exposure to 10 ng/L TPTCl in rockfish (*Sebastiscus marmoratus*), and there was testicular vacuolization at 48 days of exposure (15).

The hormones associated with reproductive control are also affected by the presence of OTs (13, 15, 26, 27). *S. marmoratus* exposed to 10 ng/L TPTCl exhibited a decrease in follicle-stimulating hormone (FSH) mRNA expression (15). In rats, exposure to 6 mg of TPTCl/kg resulted in increased levels of luteinizing hormone (LH) (13). However, mice exposed for 3 days at 0.05 and 0.5 mg/kg TBTCl exhibited a reduction in serum LH levels of approximately 50% on PND 84 (26). In other studies, rats and hamsters exposed to TBTCl at 15 mg/kg/30 days and 100–150 ppm/kg/65 days, respectively, presented a reduction in testosterone levels (13, 24). By contrast, testosterone levels increased in the treatment of frogs (*Xenopus laevis*) with 0.5 µg/L of azocyclotin (27). In addition, studies have shown a reduction in serum estrogen levels and/or testes weight upon exposure to various OTs (TBTC1, TPTC1, or azocyclotin) in different rodent, toad and fish species (15, 20, 26, 27).

### Female Reproductive Function

Organotins can also affect the female reproductive function in different animal models (12, 16, 19, 28–30). Rats exposed to 100 ng/kg of TBTCl have abnormalities in the estrous cycle and present increased ovary and serum tin levels (14, 31). In addition, OTs led to a decrease in the weight of the reproductive organs of rodents in a dose-dependent manner (21, 32–35). By contrast, Grote et al. (29) found an increase in rat ovarian weight when exposed to doses of 2–6 mg/kg/day of TPTCl for 30 days. Ma et al. (24), when exposing zebra fish to 0.09 and 0.45 µg/L of azocyclotin for 21 days, reported a reduction in the gonadosomatic index. Li et al. (27) demonstrated that adult frogs exposed to 0.05 and 0.5 g/L azocyclotin for 28 days also presented a reduction in the gonadosomatic index. The study also reported an increase in the number of hermaphroditic frogs after exposure to azocyclotin (27).

Furthermore, it has been reported that exposure to OTs causes impairment of the release and production of sex hormones (14, 24, 27, 29, 33). Grote et al. (29) demonstrated that rats treated with 6 mg/kg of TPTCl had increased serum estrogen levels. By contrast, other studies have shown a reduction in the serum estrogen levels and increased testosterone in TBTCl-treated rats (14, 27, 31, 36). Ma et al. (24) demonstrated that azocyclotin treatment caused an increase in testosterone levels and a decrease in estrogen levels in the ovaries of female zebra fish. It is also known that these xenobiotics cause abnormalities in uterine and ovarian morphology, impairing ovarian follicular development and increasing the number of atretic ovarian follicles in rodents (14, 31). Lee et al. (34) treated rats with 1–10 mg/kg bw of TBTAc for 7 days and observed an increase in ovarian follicular apoptosis. Shen et al. (37), by administering TPTCL *in vivo* (female mice: 5 or 10 mg/kg/day by oral gavage for 10 days) and *in vitro* (germinal vesicle oocytes: 100 mg/mL/1), found impairment in oocyte development *in vitro* and a reduction in the number of secondary and mature ovarian follicles *in vivo*. In zebra fish treated with azocyclotin, the development of the oocyte was also impaired (24). Thus, OT exposure impairs ovarian function in vertebrates, possibly leading to a loss of fertility.

## Fertility

Exposure to OTs in vertebrates negatively affects fertility, impairing major reproductive indicators such as pre- and postimplantation, the number of live pups, litter size, and so on (14, 38, 39). Studies have shown that female rats exposed to 7.6 and 15.2 mg/kg TBTCl presented greater pre- and postimplantation loss and a reduction in bw and the number of live fetuses in the treated groups (40). In addition, female rats exposed to 20 mg/kg TBTCl at gestational days (GDs) 0–19 showed a significant increase in postimplantation loss (41). In the same model, female rats exposed to 15.2 and 30.4 mg/kg DBTCl for 3 days showed an increase in pre- and postimplantation embryo loss (42). This embryonic/fetal loss was also observed in cynomolgus monkeys (*Macaca fascicularis*) exposed *in utero* to 2.5 and 3.8 mg/kg of DBTCl by the organogenesis period but with no effects on morphological development (43).

Monkeys exposed to 2.5 and 3.8 mg/kg DBTCl for 30 days did not show any differences in the sex ratio (43). In female rats, exposure to 20 mg/kg TBTCl reduced the litter size and increased fetal numbers. However, the sex ratio did not show significant differences *in utero* exposure (41). Data show that the exposure of Swiss mice to 1.875, 3.75, or 7.5 mg/kg/day TPTCl did not result in changes to the litter size (39). Studies with the *Oryzia latipes* fish model showed that, when exposed to a diet of 5 and 25 µg/g TBTCl for 3 weeks, the fish produced eggs with a reduced hatch capacity. However, no differences were observed in the sex ratio (44). In another study, using the same fish species but with exposure to TPTCl at different levels, reductions in the female birth rate and the number of eggs were observed for each female. In addition, the incubation capacity decreased, and many embryos died before hatching due to developmental defects (45). However, zebra fish exposed to TBT at 1 µg/g of diet showed no difference in hatchability and egg viability; however, a decrease in fecundity was observed, and the proportion of females was significantly higher (19). By contrast, zebra fish kept in tanks with a continuous flow of TBT of 0.1 ng/L from the post-hatch days 0–70 had a higher proportion of males (23).

## Placental Assessment

Several studies have shown that placental functions are also affected by the toxicological actions of OTs (Table 2) (46–49). In a human placental tissue collected between 1997 and 2001 from Finland (Turku) and Denmark (Copenhagen), relatively infrequent detection of MBT (percentage of samples > limit of quantification (LOQ) ranging from 10 to 11%) and more frequent detection of TBTCl and TPTCl (percentage of samples > LOQ ranging from 31 to 99%) were reported. The levels of di- and triorganotins in the placental samples collected from Finland were higher than in the placental samples collected from Denmark, especially for TBTCl (99 versus 37%, respectively) (48, 49). Cooke et al. (50) found 650 ng/g TBTCl in the placenta of female rats exposed to 10 mg/kg bw/day and on GD 20, and the TBTCl levels in the placenta were approximately 5-fold higher than the levels in maternal blood and 10-fold higher than in the milk on PND 6.

**Table 2:** Summary of placental changes induced by OTs.

	Animal models/dose/OTs									
	Rodents		Human				JAr and JEG-3 cells			Reference
	(100 ng to 125 mg/kg)		(0.25–20 mg/kg or 6.2 µM)				(1–100 nM)			
	TriOTs	DiOTs	TriOTs	DiOTs	TeBT	MBT	TriOTs	DiOTs	MBT	
**Placenta**										
Sn accumulation	↑	NR	↑	NR	NR	↑	NR	NR	NR	(48–50)
Aromatase activity	NR	NR	↓	↓	↔	↔	↑	↑/↔	NR	(28, 46, 47)
3β-HSD activity	NR	NR	↓	↔	↔	↔	NR	NR	NR	(46)
hCG secretion	NR	NR	NR	NR	NR	NR	↑	↑/↔	NR	(28, 47)
RXR activation	NR	NR	NR	NR	NR	NR	↑	NR	NR	(47)
17β-HSD I activity	NR	NR	NR	NR	NR	NR	↑	↑	NR	(51)
Progesterone	NR	NR	NR	NR	NR	NR	↑	↑	↑	(52)

*OTs, organotins; TriOTs, triorganotins (tributyltin chloride, tributyltin acetate, triphenyltin chloride, triphenyltin hydroxide); DiOTs, diorganotins (dibutyltin chloride, dibutyltin dichloride, diphenyltin dichloride); TeBT, tetrabutyltin; MBT, monobutyltin; Sn, tin; ↑, increased; ↓, decreased; ↔, unchanged or similar to control; NR, not reported; Rodents, rats and mice; Human, placenta samples were obtained as reported in Ref. (46, 48, 49); JAr and JEG-3 cells, human choriocarcinoma cell lines*.

Heidrich et al. (46) suggest that OTs alter the enzymes of the placental steroidogenic pathway. TBTCl was found to be a partial competitive inhibitor of human placenta cytochrome P450 aromatase activity with an IC_50_ value of 6.2 µM. The residual activity of TBTCl-saturated aromatase was 37%. DBTCl acted as a partial but less potent inhibitor of activity (65% residual activity), whereas TeBT and MBT had no effect. By contrast, human 3β-HSD (3β-hydroxysteroid dehydrogenase) type I activity was only moderately inhibited by TBTCl (80% residual activity) (46).

In the human choriocarcinoma cell line (JAR cells) used as a placental experimental model, the findings on aromatase were contrary to those by Heidrich et al. (46). TBTCl and TPTCl at a nontoxic level of 10^−7^ M for 48 h caused, through a cAMP-independent pathway, a dose-related increase in human chorionic gonadotrophin (hCG) secretion and an increase in aromatase activity; furthermore, this augmentation in enzymatic activity occurred concurrently with increases in mRNA expression and estrogen biosynthesis from androstenedione (28). Otherwise, neither of the mono-alkyltin compounds altered hCG production or aromatase activity (47). DBTCl2 stimulated aromatase activity at 30 nM but failed to induce hCG production. By contrast, DPTCl2 stimulated hCG production at 30 nM but not aromatase activity (47). Moreover, the changes in hCG and aromatase mRNA expression were nearly parallel to those in hCG secretion and aromatase activity (47).

These placental factors are both induced by specific ligands of retinoid X receptors (RXRs) (47). The treatment of an RXRα-transfected human choriocarcinoma cell line (JEG-3 cells) with 1–100 nM TBTCl for 24 or 48 h stimulated luciferase (LUC) expression from 1.5- to 9-fold, and exposing the cells to the same concentrations of TPTOH induced LUC expression from 1.8- to 19-fold, suggesting that low doses of these OTs activate RXR (47). The peroxisome proliferator-activated receptor gamma (PPAR-γ) ligand failed to increase the mRNA expression of aromatase in JAr cells, suggesting that PPAR–RXR is not involved in OTs-induced aromatase expression in the human placenta and that the RXR homodimer may be required for OTs-induced aromatase expression (47). By contrast, PPAR agonists, in addition to RXR agonists, stimulate mRNA expression of hCG, indicating that OTs-induced hCG expression might involve either PPAR–RXR heterodimers or RXR homodimers (47).

Exposure for 48 h to 100 nM of each OTs (TBTCl, TPTOH, and TPTCl) caused an increase in 17β-HSD I activity in JAr cells. TBTCl and TPTCl metabolites also altered 17β-HSD I activity, but the level of activation decreased in proportion to the dealkylation or dearylation (mono- < di- < tri-) in JAr cells. The OTs that enhanced the catalytic activity of 17β-HSD I also increased its mRNA expression. However, the mRNA effects were much more pronounced than the changes in catalytic activity (51). Exposure at the same levels and time to TBTCl and TPTCl enhanced progesterone production in JAr cells (52). TBTCl and TPTCl metabolites also altered progesterone production. However, TeBT failed to stimulate this placental function at doses of <100 nM (52). Taken together, placental OT levels and hormonal changes should reflect the abnormal placental function, and these irregularities could be associated with the abnormal development and fetal exposure levels.

## Generational Effects

Intrauterine exposure to TBTCl at different doses and routes did not alter the male:female ratio of pups in rats (50, 53). However, the exposure of zebra fish to 1 µg of TBTCl/g *via* the diet increased the proportion of females (19). Furthermore, in rats, gestational exposure to 125 ppm TBTCl did not alter the process of the descent of the male testis (12); nevertheless, in humans in Denmark, a positive correlation of the levels of DBTCl in the placenta with the occurrence of cryptorchidism in newborns was found (48). Furthermore, preputial separation in mice was not altered when exposed to 1, 10, and 100 µg/kg of TBTCl *in utero* (54).

Exposure to TBTCl by the diet with 5, 25, and 125 ppm of TBTCl/g of chow in the gestational period (GP) of rats induced a reduction in the epididymis, prostate, and testis weights in a dose-dependent manner (12). However, no significant changes in the weight of the male mouse sex organs were observed after exposure to 1, 10, and 100 µg/kg TBTCl (54), demonstrating different susceptibility/sensitivity to OTs according to the exposure model.

Studies have shown that testes can be a target organ for OTs action, as can be observed in the histological irregularities (12, 54, 55). Rat fetal testes exposed to 20 mg/kg TBTCl showed reduced numbers of gonocytes, Sertoli cells, and Leydig cells. In addition, there are differences in the expression of connexin 43 in Leydig cells, which may be reduced or completely absent (55). Moreover, seminiferous epithelial vacuolization, the retention of spermatids in the epithelium, and the retardation of spermatid maturation were observed in adult rats (12), while in mice, the sloughing of germ cells was observed in the seminiferous tubules (54); both were exposed to TBTCl in the GP.

In the parameters for sperm, OT–GP exposure has dose-dependent toxicological effects that vary according to the model of exposure used. In rats exposed to TBTCl *via* the diet, the spermatid and sperm counts were reduced, but no morphological changes or reduction in sperm motility were observed (12). In mice exposed to TBTCl in the GP until weaning, a dose-dependent reduction in sperm count was observed on both PND 49 and PND 152. A dose-dependent reduction in sperm motility at doses of 10 and 100 µg of TBTCl/kg bw (54) was also observed.

Rats exposed to TBTCl showed a dose-dependent increase in serum testosterone and LH levels as well as a reduction in the serum estrogen levels only in animals exposed to 125 ppm of TBTCl (12). In addition, mice exposed to a TBTCl dose of 10 mg Sn/kg in GD15 showed increased expression of the LH β-subunit mRNA (53). Meanwhile, mice exposed to TBTCl from the GP until weaning showed a reduction in intratesticular estrogen levels only on PND 49 (54). The most disturbing effects were observed in humans, where the LH levels in 4-month-old boys had a negative correlation while the inhibin B levels correlated positively with the levels of TBTCl in the placenta of women from Finland (48).

The effects of GP on OTs in the reproductive system of mammalian females have been underestimated until the present. Ogata et al. (56) reports that F1 and F2 generations of rats with a whole-life dietary concentration of 125 ppm of TBTCl showed a delay of approximately 6 days for vaginal opening and an impaired estrous cycle. In mice exposed to 10 or 100 µg of TBTCl/kg bw/day from GD 6 of pregnancy through the period of lactation, female offspring showed early vaginal opening and first day in estrus, thus presenting early puberty (26). In the same study, the animals showed no alteration in the weight of the female sex organs or hormonal levels. However, the animals showed a prolongation of the estrus and diestrus phases and irregularities in the estrous cycle (26). Intrauterine exposure to TBTCl at doses of 10 and 20 mg/kg bw altered the fetal ovarian morphology of rats with reduced germ cell numbers and increased apoptotic cells (55).

## Conclusion

Organotins induce endocrine-disrupting effects in vertebrates, including humans, mainly by the exposure to OT-contaminated seafood intake. The effects of OTs have been associated with gender-specific changes in the morphological functioning of reproductive organs, including gonadal cell dysfunction and weight variation in the sex organs. Moreover, OTs are capable of crossing the placental barrier and thus accumulate in the placenta and in fetal tissues, generating congenital abnormalities. The toxicity level of OTs in various species may be related to their concentration and the timing or period of life of exposure. Thus, toxicological and bioavailability studies are needed for regulatory agencies to make informed decisions about the safety of OTs in food and for the environment in general.

## Author Contributions

The topics of the article were divided among the authors JA, PP, EM, IS, CC, ON, RF, LL, JG, who contributed with research and writing. In addition, JA and PP oversaw, assemble, and review the article. JA and PP contributed equally to the study.

## Conflict of Interest Statement

The authors declare that the research was conducted in the absence of any commercial or financial relationships that could be construed as a potential conflict of interest.

## References

[1] Ludgate JW Economic and technological impact of TBT legislation on the USA marine industry. Proc Ocean Int Work Conf (1987) 4:1309–13.

[2] Hoch M Organotin compounds in the environment—an overview. Appl Geochem (2001) 16:719–43. 10.1016/S0883-2927(00)00067-6

[3] Fent K. Ecotoxicology of organotin compounds. Crit Rev Toxicol (1996) 26:1–117. 10.3109/10408449609089891 8833456

[4] Gadd GM. Microbial interactions with tributyltin compounds: detoxification, accumulation, and environmental fate. Sci Total Environ (2000) 258:119–27. 10.1016/S0048-9697(00)00512-X 11007284

[5] Colborn T Vom Saal FS Soto AM. Developmental effects of endocrine-disrupting chemicals in wildlife and humans. Environ Health Perspect (1993) 101:378–84. 10.1016/0195-9255(94)90014-0 8080506 PMC1519860

[6] USEPA – United States Environmental Protection Agency. Endocr Disruptor Screen Test Advis Committee. Washington, DC (2000). Available from: https://www.epa.gov/endocrine-disruption/endocrine

[7] Toyoda M Sakai H Kobayashi Y Komatsu M Hoshino Y Horie M Daily dietary intake of tributyltin, dibutyltin, triphenyltin and diphenyltin compounds according to a total diet study in a Japanese population. J Food Hyg Soc Jpn (2000) 41:280–6. 10.3358/shokueishi.41.280

[8] Rantakokko P Kuningas T Saastamoinen K Vartiainen T. Dietary intake of organotin compounds in Finland: a market-basket study. Food Addit Contam (2006) 23:749–56. 10.1080/02652030600779908 16807202

[9] Jadhav S Bhosale D Bhosle N. Baseline of organotin pollution in fishes, clams, shrimps, squids and crabs collected from the west coast of India. Mar Pollut Bull (2011) 62:2213–9. 10.1016/j.marpolbul.2011.06.023 21820681

[10] Kucuksezgin F Aydin-Onen S Gonul LT Pazi I Kocak F. Assessment of organotin (butyltin species) contamination in marine biota from the Eastern Aegean Sea, Turkey. Mar Pollut Bull (2011) 62:1984–8. 10.1016/j.marpolbul.2011.06.020 21764084

[11] Lee CC Hsu YC Kao YT Chen HL. Health risk assessment of the intake of butyltin and phenyltin compounds from fish and seafood in Taiwanese population. Chemosphere (2016) 164:568–75. 10.1016/j.chemosphere.2016.08.141 27632793

[12] Omura M Ogata R Kubo K Shimasaki Y Aou S Oshima Y Two-generation reproductive toxicity study of tributyltin chloride in male rats. Toxicol Sci (2001) 64:224–32. 10.1093/toxsci/64.2.224 11719705

[13] Grote K Stahlschmidt B Talsness CE Gericke C Appel KE Chahoud I. Effects of organotin compounds on pubertal male rats. Toxicology (2004) 202:145–58. 10.1016/j.tox.2004.05.003 15337578

[14] Sena GC Freitas-Lima LC Merlo E Podratz PL de Araújo JFP Brandão PAA Environmental obesogen tributyltin chloride leads to abnormal hypothalamic–pituitary–gonadal axis function by disruption in kisspeptin/leptin signaling in female rats. Toxicol Appl Pharmacol (2017) 319:22–38. 10.1016/j.taap.2017.01.021 28161095

[15] Sun L Zhang J Zuo Z Chen Y Wang X Huang X Influence of triphenyltin exposure on the hypothalamus–pituitary–gonad axis in male *Sebastiscus marmoratus*. Aquat Toxicol (2011) 104:263–9. 10.1016/j.aquatox.2011.04.018 21641294

[16] Zhang J Zuo Z Zhu W Sun P Wang C. Sex-different effects of tributyltin on brain aromatase, estrogen receptor and retinoid X receptor gene expression in rockfish (*Sebastiscus marmoratus*). Mar Environ Res (2013) 90:113–8. 10.1016/j.marenvres.2013.06.004 23850073

[17] Peranandam R Palanisamy I Lourdaraj AV Natesan M Vimalananthan AP Thangaiyan S TBT effects on the development of intersex (Ovotestis) in female fresh water prawn *Macrobrachium rosenbergii*. Biomed Res Int (2014) 2014:412619. 10.1155/2014/412619 25121096 PMC4119913

[18] Revathi P Iyapparaj P Arockia Vasanthi L Munuswamy N Arun Prasanna V Pandiyarajan J Influence of short term exposure of TBT on the male reproductive activity in freshwater prawn *Macrobrachium rosenbergii* (De Man). Bull Environ Contam Toxicol (2014) 93:446–51. 10.1007/s00128-014-1332-4 25016935

[19] Lima D Castro LFC Coelho I Lacerda R Gesto M Soares J Effects of tributyltin and other retinoid receptor agonists in reproductive-related endpoints in the Zebrafish (*Danio rerio*). J Toxicol Environ Health A (2015) 78:747–60. 10.1080/15287394.2015.1028301 26090559

[20] Chen Y Zuo Z Chen S Yan F Chen Y Yang Z Reduction of spermatogenesis in mice after tributyltin administration. Toxicology (2008) 251:21–7. 10.1016/j.tox.2008.06.015 18687377

[21] Mello MSC Delgado IF Favareto APA Lopes CMT Batista MM Kempinas WDG Sexual maturation and fertility of mice exposed to triphenyltin during prepubertal and pubertal periods. Toxicol Rep (2014) 2:405–14. 10.1016/j.toxrep.2014.12.006 28962375 PMC5598530

[22] Mitra S Srivastava A Khandelwal S. Long term impact of the endocrine disruptor tributyltin on male fertility following a single acute exposure. Environ Toxicol (2017) 32(10):2295–304. 10.1002/tox.22446 28707438

[23] McAllister BG Kime DE. Early life exposure to environmental levels of the aromatase inhibitor tributyltin causes masculinisation and irreversible sperm damage in zebrafish (*Danio rerio*). Aquat Toxicol (2003) 65:309–16. 10.1016/S0166-445X(03)00154-1 13678849

[24] Ma YN Cao CY Wang QW Gui WJ Zhu GN Effects of azocyclotin on gene transcription and steroid metabolome of hypothalamic–pituitary–gonad axis, and their consequences on reproduction in zebrafish (*Danio rerio*). Aquat Toxicol (2016) 179:55–64. 10.1016/j.aquatox.2016.08.006 27571716

[25] Kanimozhi V Palanivel K Kadalmani B Krikun G Taylor HS Apolipoprotein E induction in syrian hamster testis following tributyltin exposure. Reprod Sci (2014) 21:1006–14. 10.1177/1933719114522519 24516040

[26] Si J Han X Zhang F Xin Q An L Li G Perinatal exposure to low doses of tributyltin chloride advances puberty and affects patterns of estrous cyclicity in female mice. Environ Toxicol (2012) 27:662–70. 10.1002/tox.21756 22362710

[27] Li S Li M Gui W Wang Q Zhu G. Disrupting effects of azocyclotin to the hypothalamo–pituitary–gonadal axis and reproduction of *Xenopus laevis*. Aquat Toxicol (2017) 185:121–8. 10.1016/j.aquatox.2017.02.010 28213302

[28] Nakanishi T Kohroki J Suzuki S Ishizaki J Hiromori Y Takasuga S Trialkyltin compounds enhance human CG secretion and aromatase activity in human placental choriocarcinoma cells. J Clin Endocrinol Metab (2002) 87:2830–7. 10.1210/jcem.87.6.8540 12050258

[29] Grote K Andrade AJM Grande SW Kuriyama SN Talsness CE Appel KE Effects of peripubertal exposure to triphenyltin on female sexual development of the rat. Toxicology (2006) 222:17–24. 10.1016/j.tox.2006.01.008 16464526

[30] Zhang J Zuo Z Xiong J Sun P Chen Y Wang C Tributyltin exposure causes lipotoxicity responses in the ovaries of rockfish, *Sebastiscus marmoratus*. Chemosphere (2013) 90:1294–9. 10.1016/j.chemosphere.2012.10.078 23153777

[31] Podratz PL Filho VSD Lopes PFI Sena GC Matsumoto ST Samoto VY Tributyltin impairs the reproductive cycle in female rats. J Toxicol Environ Health A (2012) 75:1035–46. 10.1080/15287394.2012.697826 22852853

[32] Wester PW Krajnc EI van Leeuwen FXR Loeber JG van der Heijden CA Vaessen HAMG Chronic toxicity and carcinogenicity of bis(tri-n-butyltin)oxide (TBTO) in the rat. Food Chem Toxicol (1990) 28:179–96. 10.1016/0278-6915(90)90006-9 2344992

[33] Grote K Hobler C Andrade AJM Grande SW Gericke C Talsness CE Sex differences in effects on sexual development in rat offspring after pre- and postnatal exposure to triphenyltin chloride. Toxicology (2009) 260:53–9. 10.1016/j.tox.2009.03.006 19464569

[34] Lee H Lim S Yun S Yoon A Park G Yang H. Tributyltin increases the expression of apoptosis- and adipogenesis-related genes in rat ovaries. Clin Exp Reprod Med (2012) 39:15. 10.5653/cerm.2012.39.1.15 22563546 PMC3341447

[35] Podratz PL Merlo E Sena GC Morozesk M Bonomo MM Matsumoto ST Accumulation of organotins in seafood leads to reproductive tract abnormalities in female rats. Reprod Toxicol (2015) 57:29–42. 10.1016/j.reprotox.2015.05.003 26050607

[36] Schoenfelder M Schams D Einspanier R. Steroidogenesis during *in vitro* maturation of bovine cumulus oocyte complexes and possible effects of tri-butyltin on granulosa cells. J Steroid Biochem Mol Biol (2003) 84:291–300. 10.1016/S0960-0760(03)00042-6 12711015

[37] Shen YT Song YQ He XQ Zhang F Huang X Liu Y Triphenyltin chloride induces spindle microtubule depolymerisation and inhibits meiotic maturation in mouse oocytes. Reprod Fertil Dev (2014) 26:1084–93. 10.1071/RD12332 23981671

[38] Grote K Hobler C Andrade AJM Grande SW Gericke C Talsness CE Effects of *in utero* and lactational exposure to triphenyltin chloride on pregnancy outcome and postnatal development in rat offspring. Toxicology (2007) 238:177–85. 10.1016/j.tox.2007.05.033 17644232

[39] Sarpa M Lopes CMT Delgado IF Paumgartten FJR. Postnatal development and fertility of offspring from mice exposed to triphenyltin (fentin) hydroxide during pregnancy and lactation. J Toxicol Environ Health A (2010) 73:965–71. 10.1080/15287391003751752 20563930

[40] Ema M Harazono A The adverse effects of dibutyltin dichloride on initiation and maintenance of rat pregnancy. Reprod Toxicol (2000) 14:451–6. 10.1016/S0890-6238(00)00095-2 11020655

[41] Adeeko A Li D Forsyth DS Casey V Cooke GM Barthelemy J Effects of *in utero* tributyltin chloride exposure in the rat on pregnancy outcome. Toxicol Sci (2003) 74:407–15. 10.1093/toxsci/kfg131 12773765

[42] Ema M Fujii S Ikka T Matsumoto M Hirose A Kamata E. Early pregnancy failure induced by dibutyltin dichloride in mice. Environ Toxicol (2007) 22:44–52. 10.1002/tox.20232 17295259

[43] Ema M Fukunishi K Matsumoto M Hirose A Kamata E Ihara T. Developmental toxicity of dibutyltin dichloride in cynomolgus monkeys. Reprod Toxicol (2007) 23:12–9. 10.1016/j.reprotox.2006.09.003 17045456

[44] Nakayama K Oshima Y Adverse effects of tributyltin on reproduction of Japanese medaka, *Oryzias latipes*. Coast Mar Sci (2008) 32:67–76.

[45] Zhang Z Hu J Zhen H Wu X Huang C Reproductive inhibition and transgenerational toxicity of triphenyltin on medaka (*Oryzias latipes*) at environmentally relevant levels. Environ Sci Technol (2008) 42:8133–9. 10.1021/es801573x 19031914

[46] Heidrich DD Steckelbroeck S Klingmuller D. Inhibition of human cytochrome P450 aromatase activity by butyltins. Steroids (2001) 66:763–9. 10.1016/S0039-128X(01)00108-8 11522339

[47] Nakanishi T Nishikawa J Hiromori Y Yokoyama H Koyanagi M Takasuga S Trialkyltin compounds bind retinoid X receptor to alter human placental endocrine functions. Mol Endocrinol (2005) 19:2502–16. 10.1210/me.2004-0397 15941851

[48] Rantakokko P Main KM Wohlfart-Veje C Kiviranta H Airaksinen R Vartiainen T Association of placenta organotin concentrations with congenital cryptorchidism and reproductive hormone levels in 280 newborn boys from Denmark and Finland. Hum Reprod (2013) 28:1647–60. 10.1093/humrep/det040 23520400

[49] Rantakokko P Main KM Wohlfart-Veje C Kiviranta H Airaksinen R Vartiainen T Association of placenta organotin concentrations with growth and ponderal index in 110 newborn boys from Finland during the first 18 months of life: a cohort study. Environ Health (2014) 13:45. 10.1186/1476-069X-13-45 24899383 PMC4061538

[50] Cooke GM Forsyth DS Bondy GS Tachon R Tague B Coady L. Organotin speciation and tissue distribution in rat dams, fetuses, and neonates following oral administration of tributyltin chloride. J Toxicol Environ Health A (2008) 71:384–95. 10.1080/15287390701801653 18246498

[51] Nakanishi T Hiromori Y Yokoyama H Koyanagi M Itoh N Nishikawa JI Organotin compounds enhance 17beta-hydroxysteroid dehydrogenase type I activity in human choriocarcinoma JAr cells: potential promotion of 17beta-estradiol biosynthesis in human placenta. Biochem Pharmacol (2006) 71:1349–57. 10.1016/j.bcp.2006.01.014 16513093

[52] Hiromori Y Yui H Nishikawa JI Nagase H Nakanishi T. Organotin compounds cause structure-dependent induction of progesterone in human choriocarcinoma Jar cells. J Steroid Biochem Mol Biol (2016) 155:190–8. 10.1016/j.jsbmb.2014.10.010 25465476

[53] Kariyazono Y Taura J Hattori Y Ishii Y Narimatsu S Fujimura M Effect of *in utero* exposure to endocrine disruptors on fetal steroidogenesis governed by the pituitary–gonad axis: a study in rats using different ways of administration. J Toxicol Sci (2015) 40:909–16. 10.2131/jts.40.909 26558472

[54] Si J Li P Xin Q Li X An L Li J. Perinatal exposure to low doses of tributyltin chloride reduces sperm count and quality in mice. Environ Toxicol (2015) 30:44–52. 10.1002/tox.21892 23913619

[55] Kishta O Adeeko A Li D Luu T Brawer JR Morales C *In utero* exposure to tributyltin chloride differentially alters male and female fetal gonad morphology and gene expression profiles in the Sprague-Dawley rat. Reprod Toxicol (2007) 23:1–11. 10.1016/j.reprotox.2006.08.014 17095186

[56] Ogata R Omura M Shimasaki Y Kubo K Oshima Y Shuji A Two-generation reproductive toxicity study of tributyltin chloride in female rats. J Toxicol Environ Health A (2001) 63:127–44. 10.1080/15287390151126469 11393799

